# *daf-31* Encodes the Catalytic Subunit of N Alpha-Acetyltransferase that Regulates *Caenorhabditis elegans* Development, Metabolism and Adult Lifespan

**DOI:** 10.1371/journal.pgen.1004699

**Published:** 2014-10-16

**Authors:** Di Chen, Jiuli Zhang, Justin Minnerly, Tiffany Kaul, Donald L. Riddle, Kailiang Jia

**Affiliations:** 1 MOE Key Laboratory of Model Animal for Disease Study, Model Animal Research Center, Nanjing Biomedical Research Institute, Nanjing University, Nanjing, China; 2 Department of Biological Sciences, Florida Atlantic University, Jupiter, Florida, United States of America; 3 Michael Smith Laboratories, University of British Columbia, Vancouver, British Columbia, Canada; Stanford University Medical Center, United States of America

## Abstract

The *Caenorhabditis elegans* dauer larva is a facultative state of diapause. Mutations affecting dauer signal transduction and morphogenesis have been reported. Of these, most that result in constitutive formation of dauer larvae are temperature-sensitive (ts). The *daf-31* mutant was isolated in genetic screens looking for novel and underrepresented classes of mutants that form dauer and dauer-like larvae non-conditionally. Dauer-like larvae are arrested in development and have some, but not all, of the normal dauer characteristics. We show here that *daf-31* mutants form dauer-like larvae under starvation conditions but are sensitive to SDS treatment. Moreover, metabolism is shifted to fat accumulation in *daf-31* mutants. We cloned the *daf-31* gene and it encodes an ortholog of the arrest-defective-1 protein (ARD1) that is the catalytic subunit of the major N alpha-acetyltransferase (NatA). A *daf-31* promoter::GFP reporter gene indicates *daf-31* is expressed in multiple tissues including neurons, pharynx, intestine and hypodermal cells. Interestingly, overexpression of *daf-31* enhances the longevity phenotype of *daf-2* mutants, which is dependent on the forkhead transcription factor (FOXO) DAF-16. We demonstrate that overexpression of *daf-31* stimulates the transcriptional activity of DAF-16 without influencing its subcellular localization. These data reveal an essential role of NatA in controlling *C. elegans* life history and also a novel interaction between ARD1 and FOXO transcription factors, which may contribute to understanding the function of ARD1 in mammals.

## Author Summary

The development of a living organism is influenced by the environmental conditions such as nutrient availability. Under starvation conditions, the *C. elegans* larvae will enter a special developmental stage called dauer larva. An insulin-like signaling pathway controls dauer formation as well as adult lifespan by inhibiting the activity of FOXO transcription factor DAF-16 that regulates expression of stress-resistant genes. Here we isolate a new gene called *daf-31*; this gene encodes a protein that regulates *C. elegans* larval development, metabolism and adult lifespan. This protein has been found in other species to be part of an enzyme that functions to modify other proteins. We show that overexpression of our newly discovered protein stimulates the transcriptional activity of DAF-16. Interestingly, abnormal regulation of human proteins similar to DAF-31 results in tumor formation. It is known that human FOXO proteins prevent tumorigenesis. Therefore, it is possible that abnormal DAF-31 activity may lead to tumor growth by reducing DAF-16 activity. Thus, the present study may not only contribute to understanding the role of a universal enzyme in controlling development, metabolism and lifespan in other organisms besides worms but may also shed light on the mechanisms of tumorigenesis in humans.

## Introduction

Animal development is a complex process that involves hierarchical gene regulatory networks and is influenced by environmental conditions. When food is abundant, the post-embryonic development of *C. elegans* consists of four larval stages (L1–L4) and the adult. During the L1 stage, environmental factors determine whether *C. elegans* molts to an L2 larva or a pre-dauer L2d larva [1]. At least three environmental cues have been defined: food supply, temperature, and a constitutively secreted dauer-inducing pheromone that signals population density [2]. The L2 larva is developmentally committed to continued growth, whereas the L2d larva can molt to a dauer larva if food is scarce and the animals are overcrowded, or to an L3 larva should conditions improve.

Mutations affecting dauer larval development include dauer-defective (*daf-d*) mutations that prevent entry into the dauer stage, and dauer-constitutive (*daf-c*) mutations that mandate entry into the dauer stage [2]. Based on epistatic relationships between *daf-c* and *daf-d* mutations, more than twenty genes controlling dauer formation have been ordered in a genetic pathway [2] representing generation of the pheromone signal [3], response by chemosensory neurons [4], [5] and transduction of the signal to other cells. Three functionally overlapping neural pathways control the developmental response to environmental cues. They involve DAF-7/TGF-ß [6], [7], DAF-11/cyclic GMP [8], and DAF-2/insulin-like [9], [10] pathways, which relay the environmental signals to a nuclear hormone receptor, DAF-12 [11], to control dauer versus non-dauer morphogenesis.

Mutations in two genes, *daf-9* and *daf-15*, lead to non-conditional formation of detergent-sensitive dauer-like larvae [12]. These mutants form dauer larvae constitutively and display some characteristics of dauer larvae formed under starvation, such as a high density of intestinal and hypodermal storage granules. *daf-9* encodes a cytochrome P450 related to those involved in the biosynthesis of steroid hormones in mammals [13], [14]; it was found to specify a step in the biosynthetic pathway for a DAF-12 steroid ligand called dafachronic acid [15]–[17]. *daf-15* encodes the *C. elegans* ortholog of Raptor [18] that is proposed to interact with *C. elegans* target-of-rapamycin kinase (LET-363/CeTOR) to control *C. elegans* larval development [18]. Both *daf-9* and *daf-15* also regulate fat metabolism and adult lifespan [13], [14], [18].

The dauer-like mutants represent a mutant class distinct from the previously defined *daf-c* and *daf-d* mutants. Unlike most *daf-c* mutants, the dauer-like mutants are not ts, and they do not complete dauer morphogenesis. The *daf-d* genes such as *daf-12* have non-conditional alleles and fail to respond to pheromone [1], but unlike the dauer-like mutants they can execute non-dauer development. The dauer-like mutants define a third class of mutants, one in which the animals are incapable of executing either complete dauer or non-dauer development.

The *daf-31* mutant was isolated in genetic screens to identify genes similar to *daf-9* and *daf-15* [17]. The overall aim of the present study was to clone the *daf-31* gene and characterize the DAF-31 function. Our genetic epistasis analysis suggests *daf-31* functions downstream of or in parallel to *daf-3*, *daf-12* and *daf-16* dauer-defective genes, and acts upstream of or in parallel to *daf-15*/*raptor*. We cloned the *daf-31* gene by positional cloning and showed that it encodes an ortholog of arrest-defective-1 protein (ARD1), the catalytic subunit of the major N alpha-acetyltransferase (NatA). Moreover, our data reveal that *daf-31* has an essential role in controlling *C. elegans* larval development, metabolism and adult longevity.

## Results

### *daf-31* mutations cause developmental larval arrest

**Figure 1:**
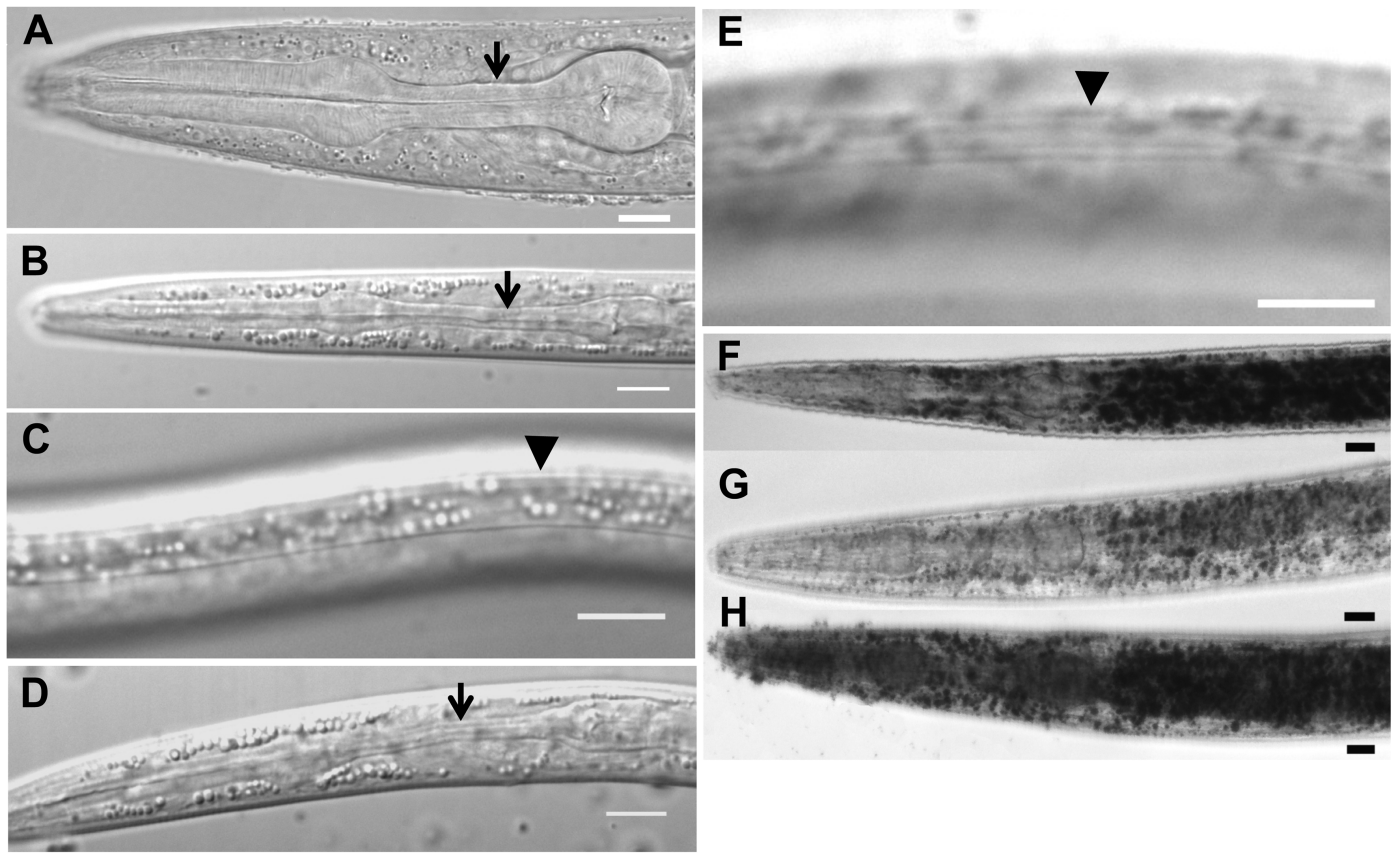
Characteristics of *daf-31* mutant dauer larvae. (A) N2 L3 larva pharynx. (B) N2 dauer larva with fully constricted pharynx. (C) N2 dauer larva with dauer alae along the lateral surface of the cuticle (body beneath focal plane). (D) *unc-24(e138)daf-31(m655)* dauer larva with fully constricted pharynx. (E) *unc-24(e138)daf-31(m655)* dauer larva with dauer alae (body beneath focal plane). Arrows indicate isthmus of pharynx in panels A, B and D. Arrowheads indicate dauer alae in panels C and E. N2 and *unc-24daf-31*/*nT1* animals were grown on NG agar plates at 20°C. Unc dauer larvae (*unc-24daf-31*) were identified after animals were starved. (F–H) Representative pictures showing fat accumulation detected by Sudan Black B in *daf-2* mutants (F), N2 (G) and *daf-31* mutants (H). N2, *daf-2(e1370)* and *daf-31(m655)IV*/*nT1*[*unc-?(n754) let-?*]*(IV;V)* synchronized L1 larvae were placed on NG agar plates, incubated at 20°C until they entered L3 or dauer-like stages, then collected for staining. Scale bars: 10 µm.

Entry into the dauer stage is determined by the pheromone/food ratio, with high pheromone and low food supply favoring dauer formation [2]. Dauer larva is considered as an alternative L3 larval stage. Compared to L3 larva (Figure 1A), the dauer larva has a constricted pharynx (Figure 1B) and a special cuticle with dauer alae (Figure 1C). In the presence of dauer-inducing pheromones, *daf-31* mutants cannot form SDS-resistant dauer larvae [17]. In order to determine whether *daf-31* mutants enter the dauer stage in response to starvation, we examined the progeny of strain *unc-24daf-31*/*nT1* under starvation conditions and observed uncoordinated (Unc) dauer larvae. These dauer larvae showed normal dauer features, such as a dark body, fully constricted pharynx (Figure 1D), and a cuticle with dauer alae (Figure 1E). However, *daf-31* dauer larvae were not SDS-resistant like normal dauer larvae. Furthermore, *daf-31* dauer larvae could not resume development when food was provided, dying shortly thereafter. Therefore, *daf-31* mutants could not complete dauer morphogenesis under starved conditions, and those incomplete dauer larvae could not finish reproductive development after food was provided.

Fat accumulation is one characteristic of *C. elegans* dauer larvae. We examined fat accumulation in *daf-31* homozygous mutants using Sudan Black B staining. As shown in Figure 1F, *daf-2* mutant dauer larvae accumulate fat as described previously [9]. The *daf-31* mutant worms also accumulate more fat droplets than wild-type worms and fat droplets in the *daf-31* mutants are larger than those in wild-type worms (Figure 1G and 1H). To confirm this phenotype, Nile red was used to stain fixed worms; this approach has been reported to reliably detect fat droplets in *C. elegans* [19]. Similar to Sudan Black staining, Nile red also detected fat accumulation in *daf-31* mutant worms (Figure S1). Therefore, *daf-31* mutants shift metabolism to fat accumulation.

### *daf-31* is epistatic to *daf-d* genes but acts upstream of or in parallel to *daf-15/raptor*

To position *daf-31* in the dauer formation pathway, we examined the epistatic relationship between *daf-31* and *daf-d* genes including *daf-3*, *daf-12* and *daf-16*. The *daf-31* mutation is epistatic to all three *daf-d* mutations as judged by the ratio of progeny (1∶2∶1) (Table 1). For the epistasis analysis with *daf-16*, the ratio of progeny is 1∶2 because *nT1* homozygous animals are lethal. We repeated the epistasis analysis of *daf-31* and *daf-12* by using the *daf-12(rh61rh411)* null allele [11] and obtained a similar result (Table 1). These epistatic relationships suggest that *daf-31* functions downstream of or in parallel to *daf-3*, *daf-12* and *daf-16* in dauer formation.

**Table 1:** Epistatic tests between *daf-31* and *daf-d* mutations for dauer formation.

Strain	Progeny (%^a^)	Ratio (N^b^)
*daf-31/unc-24; daf-3(e1376)*	dauer-like (22.2%) WT (52.4%) Unc (25.4%)	dauer: WT: Unc = 1: 2.4: 1.1 (2874)
*daf-31/unc-24; daf-12(m20)*	dauer-like (24.7%) WT (51.4%) Unc (23.9%)	dauer: WT: Unc = 1: 2.1: 0.9 (1294)
*daf-31/unc-24; daf-12(rh61rh411)*	dauer-like (22.9%) WT (50.7%) Unc (26.4%)	dauer: WT: Unc = 1: 2.2: 1.1 (3083)
*unc-24daf-31/nT1+*control vector RNAi	dauer-like Unc (33.2%) WT (66.8%)	dauer: WT = 1: 2 (3516)
*unc-24daf-31/nT1+daf-16 RNAi* c	dauer-like Unc (33.8%) WT (66.2%)	dauer: WT = 1: 2 (3847)

a Percentage of total animals scored.

b Total number of animals scored.

c *daf-16* RNAi fully suppressed dauer formation of *daf-2(e1370)* mutants at 25°C (all 3152 *daf-2* mutants treated with control vector RNAi formed dauer larvae. By contrast, only two out of 3278 *daf-2* mutants treated with *daf-16* RNAi entered dauer stage).

To examine the epistatic relationship of *daf-31* and *daf-15*, we injected dsRNA of *daf-15* into *unc-24daf-31/nT1* young adult worms. Wild-type (N2) animals were treated equally and used as controls. We examined the phenotype of progeny reproduced at various time periods after injection. The progeny reproduced between seven and eighteen hours after injection arrested development at a dauer-like stage three days after egg lay (Table 2). These dauer-like animals have a similar phenotype to *daf-15* mutants. Thus, regarding dauer entry, it appears that the *daf-15* mutation is epistatic to the *daf-31* mutation. We scored the recovery of both N2 and *unc-24daf-31* dauer-like animals two days after dauer-like arrest. 48% of N2 dauer-like worms remained at dauer-like stage and the rest of the animals recovered and grew to L4 larval or adult size (Table 2). By contrast, 100% of *unc-24daf-31* animals stayed at dauer-like stage (Table 2). For *unc-24daf-31* dauer-like larvae without *daf-15* RNAi treatment, the majority of these animals died within five days. However, surviving animals all grew to L4 larval or adult size (n = 52). Taken together, these data indicate that *daf-15* is epistatic to *daf-31* as *unc-24daf-31* mutants treated by *daf-15* RNAi form dauer-like larvae similar to *daf-15* mutants. Moreover, these two mutants have a synergistic effect on *C. elegans* development because no *unc-24daf-31* dauer-like larvae treated by *daf-15* RNAi recovered. Thus, these two genes may function in the same pathway and *daf-31* is upstream of *daf-15*. However, the possibility that these two genes act in parallel cannot be excluded.

**Table 2:** Epistatic test between *daf-31* and *daf-15* mutations for dauer recovery.

Strain	dauer (%^a^)		N^b^
	Day 3	Day 5	
N2+*daf-15* RNAi	100%	48.3%	578
*unc-24daf-31+daf-15* RNAi	100%	100%	55

a Percentage of total animals scored.

b Total number of animals scored (reproduced between seven and eighteen hours after *daf-15* dsRNA injection).

### *daf-31* encodes an ortholog of ARD1

A positional cloning strategy was used to identify the *daf-31* gene on chromosome IV between *unc-24* and *fem-3* (Figure S2A). *daf-31* was found to lie between the physical SNP markers *T09A12* and *F17E9* (Figure S2A). A genomic fragment corresponding to the K07H8.3 open reading frame fully rescued the genetic *daf-31* null mutant [*daf-31*(*m655*)] phenotype, i.e. the transgenic animals did not form dauer-like larvae, but grew to fertile adults. Sequence analysis of the *daf-31* gene in the mutant revealed a 393 bp deletion which removed 151 bp of promoter upstream of the ATG start codon and 242 bp of *daf-31* coding region downstream of the ATG start codon, which may completely block *daf-31* transcription as both the essential promoter region and the N-terminal portion of the gene were deleted (Figure S2B). Primers were designed to flank the deletion region and PCR analysis of mutant worms' genomic DNA detected a 1,449 bp band (393 bp smaller than the wild-type band) in both homozygous and heterozygous *daf-31(m655)* mutant worms (Figure S2C).

The *daf-31* gene encodes the ortholog of ARD1 with a predicted molecular weight of 21.2 kDa. ARD1 is the catalytic subunit of NatA that catalyzes the acetylation of proteins beginning with Met-Ser, Met-Gly and Met-Ala [20]. Amino acid sequence alignment showed that DAF-31 shares 75% identity with human ARD1, 77% identity with mouse ARD1, 72% identity with *Drosophila melanogaster* ARD1 and 46% identity with yeast ARD1 (Figure S2D).

Given that there is only a single mutant allele of *daf-31*, we used RNAi to inhibit *daf-31* in the N2 background to confirm the *daf-31* mutant phenotype. Inhibition of *daf-31* by feeding animals with *E. coli* that express *daf-31* dsRNA did not induce a dauer-like phenotype. From our previous work, dsRNA injection can create a stronger mutant phenotype similar to that of a genetic null mutant [18]. In vitro synthesized *daf-31* dsRNA was injected into gonads of N2 young adult worms and the progeny displayed a dauer-like phenotype similar to the *daf-31(m655)* mutants. The starvation-induced dauer morphology of *daf-31(m655)* mutants, such as dauer alae formation and contrasted pharynx (described in Figure 1) could not be examined using this RNAi method. Therefore, we examined fat accumulation in *daf-31* RNAi-treated animals. Similar to the *daf-31(m655)* mutant, *daf-31* RNAi-treated animals accumulated fat as detected by both Sudan Black and Nile red staining of fixed animals (Figure S3). Based on these results, we conclude that the *daf-31* mutant phenotypes described in this study most likely resulted from *daf-31* mutation instead of secondary mutations in the background.

### *daf-31* is expressed in multiple tissues

**Figure 2:**
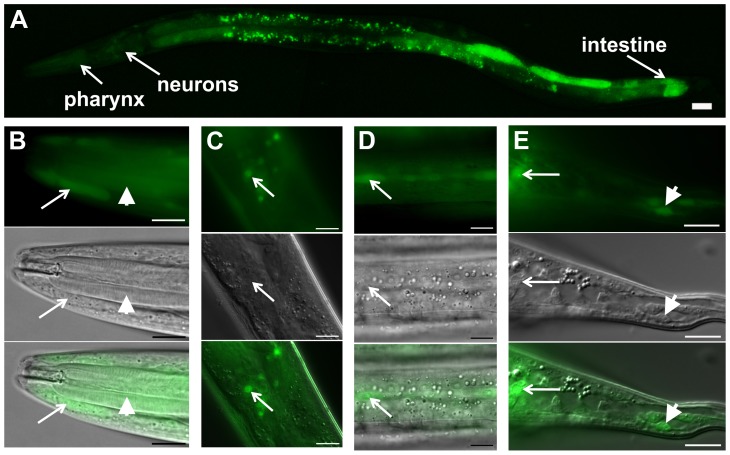
*daf-31* expression pattern in wild-type N2 animals. GFP expression is under the control of the same 760 bp *daf-31* promoter that successfully drove *daf-31* gene expression and rescued the dauer-like larval arrest phenotype of *daf-31* mutants. (A) *daf-31* expression in multiple tissues including pharynx, hypodermis, neurons and intestine. High magnification pictures showing the expression of *daf-31* in pharynx (B, indicated by arrowheads), head hypodermal cells (B, indicated by arrows), head neurons (C), hypodermal seam cells (D), tail neurons (E, indicated by arrows) and tail hypodermal cells (E, indicated by arrowheads). Photos in B though E: the upper panels show the GFP signal, the middle panels show the same animals in the same focal planes under Nomarski optics, and the bottom panels show the merged images from the upper and middle panels. For all pictures, the left is anterior and the right is posterior. Scale Bars: 10 µM.

In order to characterize the *daf-31* expression pattern, we constructed a *daf-31 promoter::gfp* reporter construct. In N2 animals, GFP expression was detected from L1 to the adult stages in multiple tissues including the hypodermis, pharynx, intestine, and neurons (Figure 2). To confirm the GFP expression pattern of *daf-31* promoter fusion reporter, we constructed *daf-31* translational fusion reporter genes in which the GFP open reading frame was fused to the full-length *daf-31* genomic DNA in frame either at the N-terminus or at the C-terminus. Both translation fusion reporter genes fully rescued the dauer-like phenotypes of *daf-31* mutants. However, we did not observe GFP expression in the rescued *daf-31* mutant worms, a phenomenon previously reported with other GFP fusion gene mutant rescues [21]. Thus, our observations of *daf-31* expression pattern were limited to the *daf-31* promoter fusion, which may not represent the endogenous expression pattern of the entire *daf-31* gene if enhancer elements are present in introns or in 3′ untranslated sequences.

### *daf-31* influences *C. elegans* lifespan

**Figure 3:**
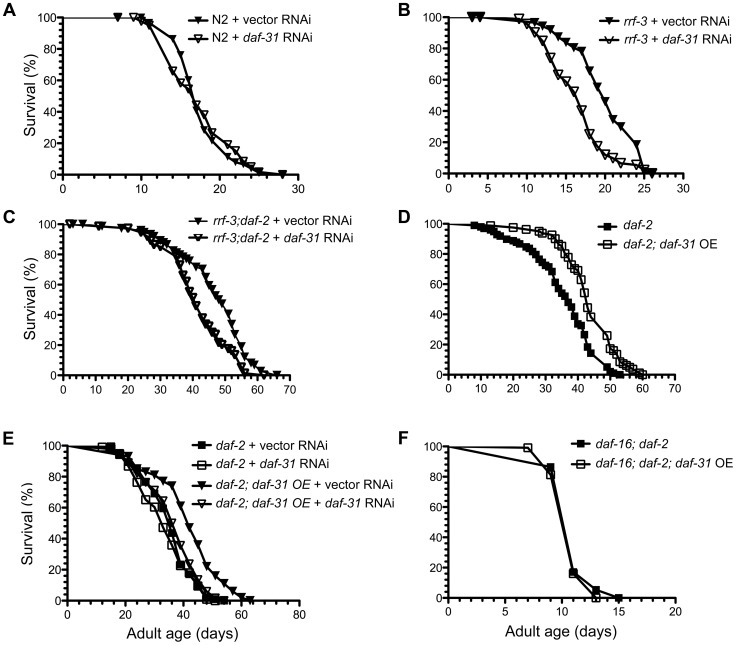
Influence of *daf-31* on *C. elegans* lifespan. (A) N2 animals treated with *daf-31* RNAi have a similar lifespan to those treated with the vector RNAi control. *daf-31* RNAi significantly decreases the lifespan of RNAi-sensitive *rrf-3* mutants (B) and RNAi-sensitive *daf-2* mutants (*rrf-3;daf-2*) (C). (D) *daf-31* overexpression enhances the longevity phenotype of *daf-2* mutants. (E) *daf-31* RNAi abrogates the effect of *daf-31* overexpression on the *daf-2* mutant lifespan. (F) *daf-16* mutations block the further lifespan extension of *daf-2* mutants conferred by *daf-31* overexpression.

Increased adult longevity is a phenotype associated with many dauer mutants. Since *daf-31* homozygous mutants arrest development at L4 stage, we inhibited the *daf-31* gene by feeding RNAi. The RNAi treatment successfully reduced *daf-31* mRNA level as measured by qRT-PCR (Figure S4). However, *daf-31* RNAi treatment had no obvious effect on the lifespan of wild-type worms as the *daf-31* RNAi-treated worms had similar mean and maximum lifespans as control vector RNAi-treated worms (Figure 3A and Table S1). When RNAi-sensitive *rrf-3(pk1426)* mutants were treated with *daf-31* RNAi, their lifespans were significantly decreased (p<0.0001, log-rank test) (Figure 3B and Table S1). Compared to controls, the mean lifespan of *rrf-3* mutants treated with *daf-31* RNAi was shortened by four days (Figure 3B and Table S1).

To test if *daf-31* mutations influence the longevity phenotype of *daf-2* mutants, we treated the *rrf-3(pk1426);daf-2(e1370)* mutant with *daf-31* RNAi. The mean lifespan of *daf-31* RNAi-treated *rrf-3;daf-2* mutants was five days shorter compared to that of control animals (p = 0.0005, log-rank test) (Figure 3C and Table S1). Thus, inhibition of *daf-31* partially suppressed the longevity phenotype of *daf-2* mutants. Based on this result, we postulate that overexpression of *daf-31* may further increase the lifespan of *daf-2* mutants.

To test this, *daf-2(e1370)* and *daf-16(mgDf47);daf-2(e1370)* mutants overexpressing *daf-31* were constructed. The overexpression of *daf-31* was confirmed by qRT-PCR (Figure S4). As shown in Figure 3D, *daf-31* overexpression increased the lifespan of *daf-2* mutant worms (p<0.0001, log-rank test) (Figure 3D and Table S1). The mean lifespan was increased by eight days and the maximum lifespan was extended by seven days (Figure 3D and Table S1). This increased lifespan was due to *daf-31* overexpression as *daf-31* RNAi treatment completely abrogated it (Figure 3E and Table S1). Interestingly, *daf-31* overexpression failed to extend the lifespan of *daf-16;daf-2* double mutants (Figure 3F and Table S1), indicating that DAF-16 is required for *daf-31* overexpression to enhance the *daf-2* longevity phenotype. We also measured the lifespan of N2 animals overexpressing the *daf-31* gene. As shown in Figure S5 and Table S1, *daf-31* overexpression did not extend the lifespan of N2 worms. In fact, it slightly decreased the lifespan of N2 worms. Finally, to confirm *daf-31* functions through *daf-16* in *C. elegans* lifespan regulation, we tested if *daf-31* RNAi can further decrease the lifespan of RNAi-sensitive *daf-16* mutants (*daf-16;rrf-3*). We found *daf-31* RNAi had no obvious effect on the lifespan of *daf-16;rrf-3* mutants (Figure S6 and Table S1).

### *daf-31* overexpression stimulates *daf-16* transcriptional activity

**Figure 4:**
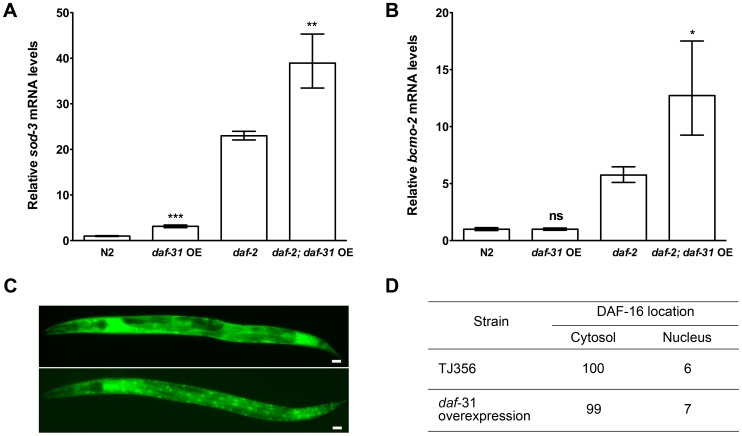
*daf-31* overexpression stimulates the transcriptional activity of DAF-16 without influencing the subcellular localization of DAF-16. qRT-PCR was performed to measure the mRNA expression level of two DAF-16 target genes, *sod-3* and *bcmo-2*, in indicated strains. Y-axis stands for relative mRNA levels. *Daf-31* overexpression up-regulates the expression of *sod-3* mRNA in both N2 worms and *daf-2* mutants (A), and up-regulates the expression of *bcmo-2* mRNA in *daf-2* mutants (B) **, P<0.01, ***, P<0.001 (*t*-test). (C) Representative pictures showing the cytosolic localization of DAF-16 (upper panel) and nuclear localization of DAF-16 (lower panel). Scale Bars: 10 µM. (D) The percentage of worms showing the nuclear localization of DAF-16 in wild-type background (TJ356) is similar to that in *daf-31* overexpression animals.

The forkhead transcription factor DAF-16/FOXO controls the transcription of an array of genes essential for lifespan extension and oxidative stress resistance including the antioxidant enzyme superoxide dismutase *(sod)-3* gene and beta-carotene 15,15′-monooxygenase gene (*bcmo-2*) [22]. We used qRT-PCR to measure the expression level of *sod-3* and *bcmo-2* in *daf-31* overexpression strains and control animals. As shown in Figure 4A, the expression level of *sod-3* was significantly increased when *daf-31* was overexpressed in the N2 background and in *daf-2* mutants. Similar to *sod-3*, the expression of *bcmo-2* is also significantly increased when *daf-31* is overexpressed in the *daf-2* mutant background (Figure 4B). Taken together, these data indicate overexpression of *daf-31* stimulates the transcriptional activity of DAF-16.

Reduction of *daf-2* insulin-like signaling activity increases *C. elegans* lifespan by promoting nuclear localization of DAF-16 [23], [24]. We crossed the DAF-16::GFP reporter gene into *daf-31* overexpressing animals to examine if *daf-31* overexpression influences the subcellular localization of DAF-16. We found the percentage of animals showing DAF-16 nuclear localization was not significantly different between *daf-31* overexpressing animals and control worms (Figure 4C and D).

### *daf-31* overexpression does not confer stress resistance

*daf-2* mutants are resistant to environmental stresses such as high temperature [25], [26]. We examined if *daf-31* overexpression could enhance the thermotolerance of *daf-2* mutants. As reported previously [25], the survival rate of *daf-2* mutants at 35° is doubled compared to N2 worms (Figure S7 and Table S2). However, the mean survival for N2 and N2 overexpressing *daf-31* was similar (9.8 hours vs. 10 hours) (p = 0.2420, log-rank test) (Figure S7 and Table S2). Similarly, *daf-31* overexpression did not increase the survival of *daf-2* mutants at 35° (p = 0.4623, log-rank test) (Figure S7 and Table S2).

### *daf-31* overexpression enhances reproduction

We tested the influence of *daf-31* overexpression on the reproduction of N2 and *daf-2* mutant animals. *daf-31* overexpression increased the brood size of N2 animals and *daf-2* mutants by about 23% and 30%, respectively (Figure S8). While *daf-31* overexpression increased the *daf-2* mutant lifespan in a *daf-16* dependent manner, *daf-31* overexpression increased the reproduction of *daf-2* mutants significantly in a *daf-16* independent way. Overexpression of *daf-31* increased the brood size of *daf-16;daf-2* mutants by 40% (P<0.01, *t*-test) (Figure S8).

## Discussion

We demonstrated that *daf-31* mutants form dauer-like larvae that share some characteristics of wild-type dauer larvae such as fat accumulation. Many *daf* genes, especially those from the insulin-like signaling pathway, are involved in the regulation of lifespan [27]. Mutations in *daf-2*, which encodes an insulin/IGF-1 receptor [9], convey a temperature-sensitive Daf-c phenotype, and the adults live twice as long as wild-type animals [9], [28], [29]. Mutations in some genes downstream of *daf-2*, such as *age-1* and *pdk-1*, also extend lifespan [30], [31]. Conversely, mutations in other downstream genes, including *daf-18* and *daf-16*, shorten lifespan [32]–[34].

We examined whether *daf-31* is also involved in aging and found that *daf-31* partially mediates the effect of reduced *daf-2/*IGF signaling pathway on *C. elegans* lifespan. Moreover, overexpression of *daf-31* enhances the longevity phenotype of *daf-2* mutants depending on the activity of DAF-16. Supporting this lifespan data, the expression levels of *sod-3* and *bcmo-2*, the transcriptional targets of the DAF-16 FOXO3 transcription factor, are up-regulated in the *daf-31* overexpression strains. Thus, it is reasonable to argue that DAF-31 regulates *C. elegans* lifespan by influencing DAF-16 transcriptional activity and *daf-31* overexpression stimulates DAF-16 activity. Indeed both DAF-31 and DAF-16 are expressed in neurons and intestine, two major tissues essential for regulation of *C. elegans* lifespan by the DAF-2/IGF signaling pathway [35]–[37]. However, overexpression of *daf-31* has no influence on the subcellular localization of DAF-16. It is consistent with the lifespan data that overexpression of *daf-31* does not increase the lifespan of N2 animals. Thus, overexpression of DAF-31 only extends *C. elegans* lifespan in the *daf-2* mutant background in which DAF-16 has entered the nucleus due to inhibition of the IGF signaling.

Previous studies show that the stress-resistance phenotype can be uncoupled from the longevity phenotype [38]. Indeed, although *daf-31* overexpression further increases the long-lived lifespan of *daf-2* mutants, it has no effect on the thermotolerance of *daf-2* mutants. Interestingly, *daf-31* overexpression increases the reproduction of both wild-type animals and *daf-2* mutants, which is not dependent on DAF-16, suggesting DAF-31 functions through DAF-16 for lifespan regulation but not for reproduction. Since DAF-16 is required for the stress resistance of *daf-2* mutants, it is likely that *daf-31* overexpression extends the *daf-2* mutant lifespan through DAF-16-dependent mechanisms other than increasing stress-resistance. DAF-31 is found in multiple tissues including neurons. It is known that many *C. elegans* neurons are refractory to RNAi treatment in wild-type background [39]. It is possible that neuronal DAF-31 activity is more important for lifespan regulation because *daf-31* RNAi treatment only shows influence on lifespan of RNAi-sensitive mutants. Supporting this assumption, it has been reported that *daf-16/FOXO* activity in neurons accounted for only 5–20% of the lifespan extension seen in *daf-2* mutants [37]. Since DAF-31 may only influence DAF-16 activity in neurons, its overexpression only increases the *daf-2* mutant lifespan modestly.

We cloned the *daf-31* gene and sequence analysis indicates DAF-31 is a worm ortholog of ARD1 that was first identified in yeast [40]. ARD1 is the catalytic subunit of the major NatA that transfers an acetyl group from acetyl coenzyme A to the N-terminal of nascent polypeptides. Yeast ARD1 mutants fail to enter stationary phase and sporulate during nitrogen deprivation [40]. The yeast stationary phase is comparable to *C. elegans* dauer stage and is essential for survival when nutrients are limited. *C. elegans* enters dauer stage during starvation or under high concentration of pheromone. Our data show *daf-31* mutants could not complete dauer morphogenesis in response to pheromone and starvation, which indicates *daf-31* is required for dauer formation. Thus, both yeast ARD1 and worm DAF-31 play an important role in the developmental switch in response to the environmental nutrient limitation. Additionally, *daf-31* mutants could not grow to fertile adults in an environment with abundant food suggesting its essential role in normal development. Similar to our observation, it has been reported that loss of *Ard1* is lethal for *D. melanogaster* and affects cell survival or proliferation, indicating ARD1 is required for *D. melanogaster* development [41]. In addition to developmental arrest, the *daf-31* mutants shift metabolism to fat accumulation. Interestingly, yeast ARD1 mutants not only fail to enter stationary phase but also do not accumulate as much carbohydrates as wild-type yeast strains [40]. Thus, the function of ARD1 in regulating development and metabolism appears conserved from yeast to *C. elegans*.

N-terminal acetylation is one of the most common posttranslational protein modifications. It is estimated to occur on 50% of yeast proteins [20], 71% of *D. melanogaster* cytosolic proteins [20] and 84% of human proteins [42]. NatA plays the most prominent role in N-terminal acetylation. It would be interesting to know whether the pleiotropic phenotypes of *ard1* mutants result from global changes of protein N-acetylation or from acetylation status of specific protein substrates. It has been reported that human ARD1 directly acetylates β-catenin and enhances its transcriptional activity [43]. We show that overexpression of DAF-31 stimulates the transcriptional activity of DAF-16. It would be interesting to examine if DAF-31 overexpression acetylates DAF-16. Alternatively, a suppressor screening of *daf-31* mutants may help to identify the essential substrates of the DAF-31 acetyltransferase and contribute to understanding the mechanisms by which ARD1 influences development, metabolism and aging. Moreover, emerging evidence has revealed that abnormal regulation of ARD1 is associated with tumorigenesis and ARD1 represents a novel cancer drug target [44], [45]. Identification of DAF-31 substrate proteins may uncover new therapeutic targets of cancer diseases.

## Materials and Methods

### Culture conditions and *C. elegans* strains

All strains were grown on NG agar plates seeded with *E. coli* strain OP50 [46]. Mutations are listed by linkage groups as follows: LG I: *daf-16(mgDf47)*; LG II: *rrf-3(pk1426)*; LG III: *daf-2(e1370)*; LG IV: *unc-24(e138)*, *daf-31(m655)*; LG X: *daf-3(e1376)*, *daf-12(m20), daf-12(rh61rh411)*. All mutants are derived from the wild-type Bristol N2 strain.

To make the *daf-16(mgDf47)I*; *daf-2(e1370)III* double mutant, *daf-2(e1370)* males were mated with *daf-16(mgDf47)* hermaphrodites. Ten F1 adults (*daf-16/+*; *daf-2/+*) were incubated at 25°C. F2 dauer larvae (either *+/+*; *daf-2* or *daf-16/+*; *daf-2*) were transferred to a fresh plate at 15°C for recovery. Then adults were shifted to 25°C. Since *daf-16(mgDf47)* can suppress the *daf-2(e1370)* Daf-c phenotype, non-dauer adults from the next generation were *daf-16(mgDf47)*; *daf-2(e1370)* double mutants.

Double mutants were constructed for epistatic tests between *daf-31* and *daf-d* mutants at 20°. However, *daf-16* RNAi was used for epistasis analysis between *daf-31* and *daf-16*. *daf-16* RNAi fully suppressed dauer formation of *daf-2(e1370)* mutants. *daf-12(m20)* and *daf-12(rh61rh411)* mutations were used to construct the strain+*daf-31(m655)/unc-24(e138)*+; *daf-12(m20)* and +*daf-31(m655)/unc-24(e138)*+; *daf-12(rh61rh411)* using standard genetic methods. The *daf-12(rh61rh411)* mutation was confirmed by sequencing. +*daf-31(m655)/unc-24(e138)*+; *daf-3(e1376)* was constructed to determine the epistatic relationship between *daf-31* and *daf-3*. As the *daf-31(m655)* mutation was not marked by a genetic mutation in *daf-31;daf-3* and *daf-31;daf-12* mutant worms, the *daf-31(m655)* deletion mutations were confirmed by using single worm PCR. Representative gel pictures are shown in Figure S9. Injection of RNAi was used to inhibit the *daf-15* gene in *unc-24(e138)daf-31(m655)/nT1* to examine the epistatic relationship between *daf-15* and *daf-31*.

To construct the *daf-31* overexpressing strains, the full-length *daf-31* genomic DNA, including its native 760-bp promoter and 3′-UTR was cloned into pGEM-T (Promega); this construct successfully rescued the *daf-31* dauer-like mutant phenotype. Multiple copies of the construct were integrated into chromosomal DNA by γ-irradiation to make an N2 transgenic line overexpressing *daf-31*. Then the *daf-31* overexpressing chromosome was introduced into both *daf-2(e1370)* and *daf-16(mgDf47);daf-2(e1370)* mutants by genetic crosses.

To make a *daf-31* promoter-GFP transcriptional fusion, the 760-bp *daf-31* promoter was inserted into the GFP vector pPD95.70 (a gift from Dr. Andrew Fire at Stanford University) between the *Pst*I and *Bam*HI sites. The construct was injected into N2 adults at a concentration of 100 µg/ml. pRF4, which encodes a mutant collagen and induces a dominant roller (Rol) phenotype, was co-injected at the same concentration as a transformation marker. Rol animals were selected from the F2 generation and used to establish stable transgenic lines.

To evaluate the subcellular location of DAF-16, TJ356 (*zIs356 [daf-16p::daf-16a/b::GFP+rol-6]*) males were crossed to *daf-31* overexpressing hermaphrodites. The roller progeny were mounted on 2% agar pads to examine DAF-16::GFP subcellular localization.

### Fat staining

To stain fat using Sudan Black B, N2, *daf-2(e1370)* and *daf-31(m655)IV*/*nT1*[*unc-?(n754) let-?*]*(IV;V)* synchronized L1 larvae were placed on NG agar plates, incubated at 20°C until they entered L3 or L4 stages, collected and washed two to three times with M9 buffer. Paraformaldehyde stock solution (10%) was added to a final concentration of 1%. The samples were frozen in dry ice/ethanol and then thawed under a stream of warm water. After a total of three freeze-thaw cycles, the worms were stained with Sudan Black B as described by Kimura et al. [9].

Nile red staining of fixed worms was performed as described by Pino et al. [47]. Worm samples were collected and washed twice with M9 buffer. After the final wash, worms were fixed in 40% isopropanol at room temperature for three minutes. The fixed worms were stained in Nile red/isopropanol solution for 30 minutes at room temperature with gentle rocking. The stained worms were washed once with 1 ml M9 buffer and mounted on a 2% agarose pad for microscopy under the fluorescence channel. In order to compare the fat content in different strains, the pictures were taken at the same camera setting under 20× magnification.

### *daf-31* cloning

Three-factor-mapping with SNP markers and cosmid rescue were performed as previously described [18]. To determine the mutation in *daf-31(m655)*, the K07H8.3 gene (GenBank accession # NM_068991.4) was amplified using primers 5′- GTG AGT CGA AAC CCA TTT TG -3′ and 5′- GAA TGA ACC AGT TGG AAA AGG -3′ from both N2 and *daf-31(m655)* mutant homozygotes. PCR products were cloned into the pGEM-T vector (Promega) following the manufacturer's instructions. T7 primer 5′- GTA ATA CGA CTC ACT ATA GGG -3′ and SP6 primer 5′- TAC GAT TTA GGT GAC ACT ATA G -3′ were used in DNA sequencing reactions.

### RNAi

Part of the *daf-31* coding region was amplified from *C. elegans* genomic DNA using primers 5′- CGG GAT CCA TTC GTT GTG CTC GCG TG -3′ and 5′- CCC AAG CTT GCA GTG GTA TAG GCC TC -3′. The PCR products were then purified and cloned into the feeding RNAi vector L4440 (Addgene) between the *Bam*HI and *Hind*III sites. The RNAi construct was transformed into *E. coli* HT115 (DE3) and RNAi feeding was performed as previously described [48].

To inhibit *daf-15* and *daf-31* genes by injection of RNAi, a 1 kb *daf-15* cDNA fragment and the full-length *daf-31* cDNA were cloned into pGEM-T vector (Promega), respectively. The gene identity was confirmed by sequencing. The Riboprobe Combination System-SP6/T7 (Promega) was used to transcribe RNA in vitro according to the manufacturer's protocol. Double-stranded RNA was synthesized and injected as described by Fire et al. [49].

### qRT-PCR

Synchronized N2 L1 larvae were treated with either control (empty) vector or *daf-31* RNAi by feeding as previously described [48]. Day 1 adult animals were collected for total RNA extraction using the Trizol kit (Zymo). Synchronized L1 larvae of *daf-31* overexpressing strains were allowed to grow on OP50 food plates. Day 1 adult animals were collected for total RNA extraction using the Trizol reagent (Zymo). The first strand cDNA was synthesized using the ImProm-II reverse transcription system (Promega). SYBR green dye (Quanta) was used for qRT-PCR to measure the expression level of *daf-31*, *sod-3* and *bcmo-2* in corresponding worm samples. Reactions were performed in triplicate on an ABI Prism 7000 real-time PCR machine (Applied Biosystems). Relative-fold changes were calculated using the 2^−ΔΔCT^ method. The primers used for qRT-PCR were: *daf-31*, 5′- GAA GAT CAC AAG GGA AAT GTT G -3′ and 5′- CTC TTG CGG TCT GAT CCA TC -3′; *act-1*, 5′- CAA TCC AAG AGA GGT ATC CTT ACC CTC -3′ and 5′- GAG GAG GAC TGG GTG CTC TTC -3′; *bcmo-2*, 5′- GCC GAT TTA GAG AAC GGA GAT CAC -3′ and 5′- TGA GAA TTC CGT CAT CTT CCC GA -3′; *sod-3*, 5′- GGA ATC TAA AAG AAG CAA TTG CTC -3′ and 5′- CGC GCT TAA TAG TGT CCA TCA G -3′.

### Adult lifespan, thermotolerance and reproduction

About 120–150 L4 larvae raised at 20°C were transferred to ten NG agar plates (twelve to fifteen animals per plate spread with either OP50 or RNAi food) and incubated at 25°C. The first day of adulthood is day 1 in the survival curves. During the reproductive period, adult animals were transferred daily to fresh plates. Thereafter, animals were transferred every ten days (OP50 food) or every six days (RNAi food). Animals were scored as alive, dead, or lost every other day. Animals that do not move in response to touching were scored as dead. Animals that died from causes other than aging, such as sticking to the plate walls, internal hatching or bursting in the vulval region, were scored as lost. GraphPad Prism was used for statistical analysis and generation of survival curves. For the thermotolerance experiment, day 1 adult animals were incubated at 35°C and survival was scored as described above. To measure reproduction of worms, L4 larvae growing at 20°C were transferred daily to fresh plates and the progeny were counted.

## Supporting Information

Figure S1 Fat accumulation in *daf-31* mutants. Nile red staining of fixed worms detects more fat droplets in *daf-2(e1370)* (A) and *daf-31* mutants (C) than those in N2 animals (B). N2, *daf-2(e1370)* and *daf-31(m655)IV*/*nT1*[*unc-?(n754) let-?*]*(IV;V)* synchronized L1 larvae were placed on NG agar plates, incubated at 20°C until they entered L3 or dauer-like stages, then collected for staining. Scale bars: 10 µm.
(TIF)

Figure S2 *daf-31* encodes an ortholog of ARD1. (A) Physical map of the *daf-31* region of chromosome IV (corresponding to 0.54 map units). (B) Schematic structure of *daf-31* genomic DNA. The closed black boxes represent exons and solid lines are introns. The open box represents the deletion in the *daf-31*(*m655*) mutant allele. (C) PCR detected a 393 bp deletion (the actual size of the lower deletion band is 1,449 bp) in *daf-31* heterozygous and homozygous mutant worms. (D) Alignment of the DAF-31 protein with its orthologs. Identical amino acids are in black boxes.
(TIF)

Figure S3 Fat accumulation in *daf-31* RNAi-treated wild-type animals. Sudan Black staining and Nile red staining of fixed worms detect more fat droplets in *daf-2(e1370)* (A and D, respectively) and *daf-31* mutants (C and F, respectively) than those in N2 animals (B and E, respectively). Scale bars: 10 µm.
(TIF)

Figure S4 *daf-31* RNAi knocks down the mRNA level of *daf-31*. qRT-PCR shows the reduced *daf-31* mRNA level in *daf-31* RNAi-treated wild-type worms and increased *daf-31* mRNA level in *daf-31* overexpressing worms. *daf-31* RNAi treatment successfully knocks down *daf-31* mRNA level in *daf-31* overexpressing worms. ***, P<0.001 (*t*-test).
(TIF)

Figure S5 *daf-31* overexpression does not extend wild-type *C. elegans* lifespan. N2 worms and *daf-31* overexpressing animals were grown at 20° in the presence of food. L4 hermaphrodites were picked up for lifespan experiments. The statistical analysis of lifespan data is presented in Table S1.
(TIF)

Figure S6 *daf-31* RNAi does not influence the lifespan of RNAi-sensitive *daf-16* mutants. *daf-16;rrf-3* animals were fed *E. coli* that express *daf-31* dsRNA or *E. coli* carrying the empty vector. The lifespan of RNAi-treated progeny were measured at 20°.
(TIF)

Figure S7 Thermotolerance of animals overexpressing *daf-31*. *daf-31* overexpression does not influence the resistance of N2 worms (p = 0.2420, log-rank test) and *daf-2* mutant adults to heat stress at 35°C (P = 0.4623, log-rank-test).
(TIF)

Figure S8 *daf-31* overexpression increases reproduction. *daf-31* overexpression increases the total number of progeny of N2 and *daf-2* mutants, and is not dependent on DAF-16. ** P<0.01, *** P<0.0001 (*t*-test).
(TIF)

Figure S9 The *daf-31* deletion mutation in *daf-31;daf-d* mutants detected by single worm PCR. Representative gel pictures showing the *daf-31* deletion mutation in all *daf-31;daf-3* (A) and *daf-31;daf-12* (B) homozygous mutants. Arrows indicate the 1,842 bp wild-type band and the 1,449 bp deletion band, respectively.
(TIF)

Table S1 Statistical analysis of *daf-31* lifespan data. *^a^* Mean lifespan for each trial. *^b^* Maximum lifespan for each trial. *^c^* Percentage of changes in mean lifespan relative to corresponding control for each trial. *^d^* Numbers of animals counted for each trial. *^e^ p* values (log-rank test) compared to corresponding control.
(DOCX)

Table S2 Statistical analysis of thermotolerance experimental data. *^a^* Mean survival for each trial. *^b^* Maximum survival for each trial. *^c^* Percentage of changes in mean survival relative to corresponding control for each trial. *^d^* Numbers of animals counted for each trial. *^e^ p* values (log-rank test) compared to corresponding control.
(DOCX)
