# Supplementary figures and images for: daf-31 Encodes the Catalytic Subunit of N Alpha-Acetyltransferase that Regulates Caenorhabditis elegans Development, Metabolism and Adult Lifespan
Source: PLoS Genet. 2014 Oct 16;10(10):e1004699. doi: 10.1371/journal.pgen.1004699 (PMC4199510; doi:10.1371/journal.pgen.1004699)

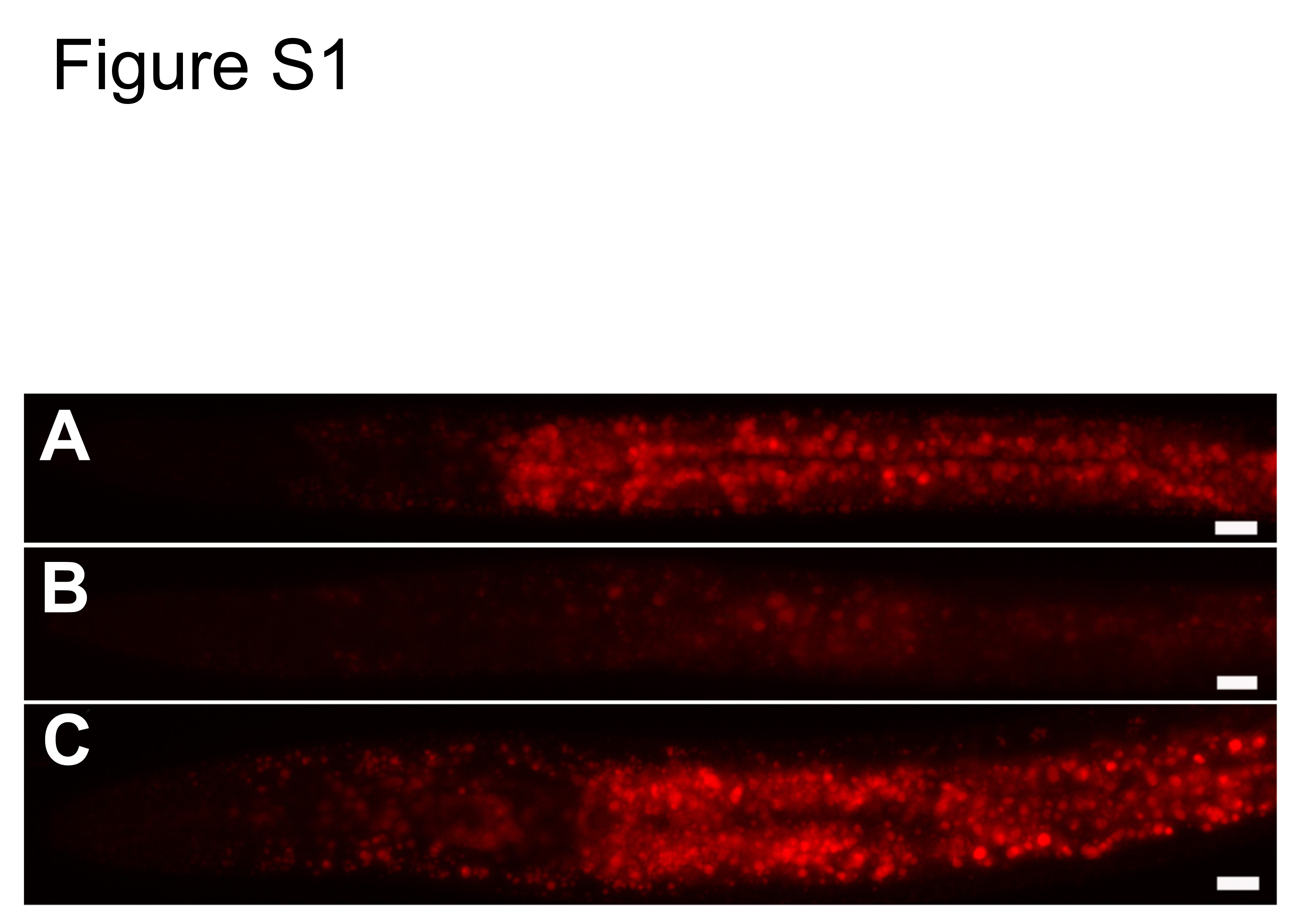

Supplement: Figure S1 — Fat accumulation in daf-31 mutants. Nile red staining of fixed worms detects more fat droplets in daf-2(e1370) (A) and daf-31 mutants (C) than those in N2 animals (B). N2, daf-2(e1370) and daf-31(m655)IV/nT1[unc-?(n754) let-?](IV;V) synchronized L1 larvae were placed on NG agar plates, incubated at 20°C until they entered L3 or dauer-like stages, then collected for staining. Scale bars: 10 µm. (TIF) [file pgen.1004699.s001.tif]

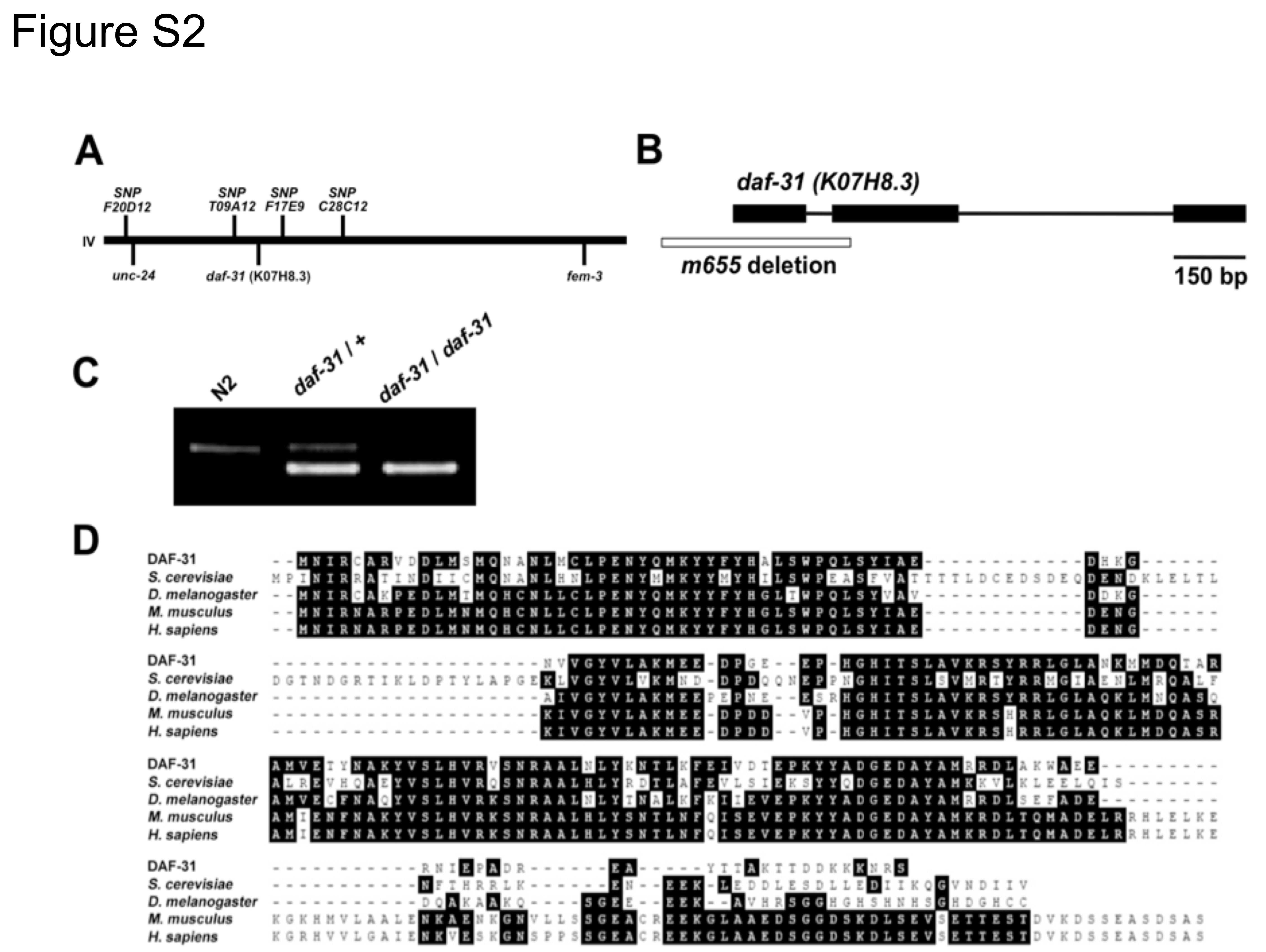

Supplement: Figure S2 — daf-31 encodes an ortholog of ARD1. (A) Physical map of the daf-31 region of chromosome IV (corresponding to 0.54 map units). (B) Schematic structure of daf-31 genomic DNA. The closed black boxes represent exons and solid lines are introns. The open box represents the deletion in the daf-31(m655) mutant allele. (C) PCR detected a 393 bp deletion (the actual size of the lower deletion band is 1,449 bp) in daf-31 heterozygous and homozygous mutant worms. (D) Alignment of the DAF-31 protein with its orthologs. Identical amino acids are in black boxes. (TIF) [file pgen.1004699.s002.tif]

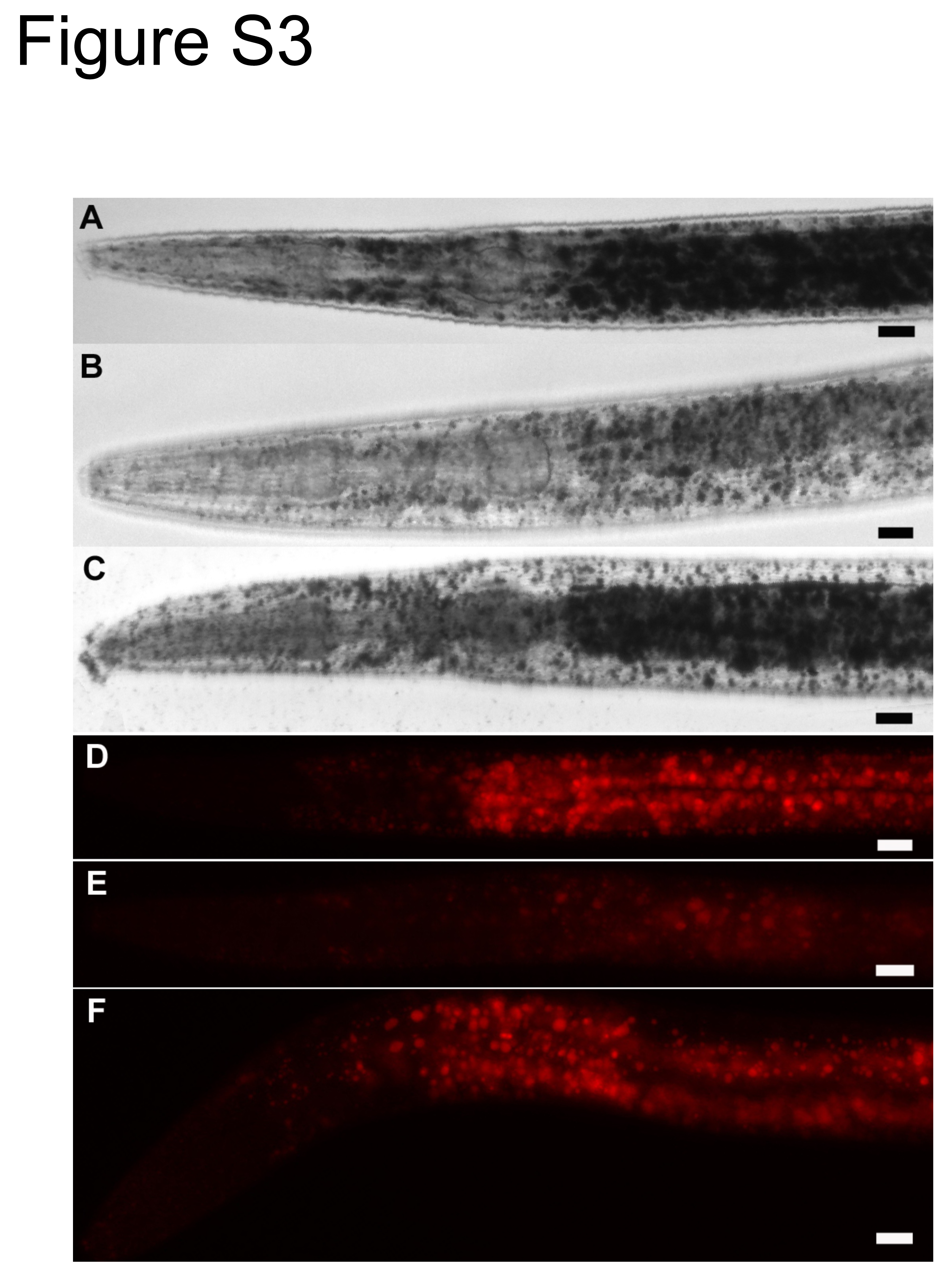

Supplement: Figure S3 — Fat accumulation in daf-31 RNAi-treated wild-type animals. Sudan Black staining and Nile red staining of fixed worms detect more fat droplets in daf-2(e1370) (A and D, respectively) and daf-31 mutants (C and F, respectively) than those in N2 animals (B and E, respectively). Scale bars: 10 µm. (TIF) [file pgen.1004699.s003.tif]

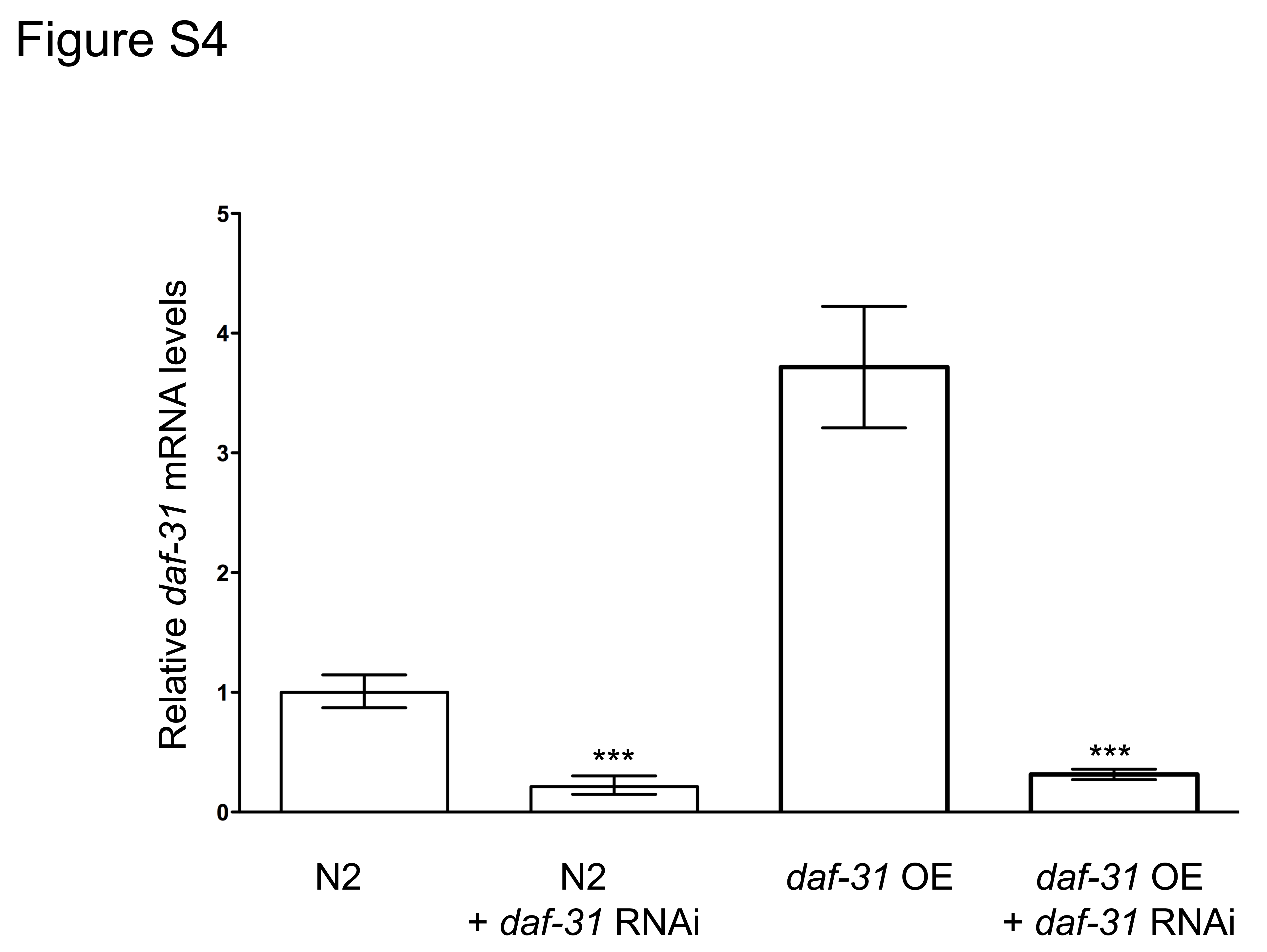

Supplement: Figure S4 — daf-31 RNAi knocks down the mRNA level of daf-31. qRT-PCR shows the reduced daf-31 mRNA level in daf-31 RNAi-treated wild-type worms and increased daf-31 mRNA level in daf-31 overexpressing worms. daf-31 RNAi treatment successfully knocks down daf-31 mRNA level in daf-31 overexpressing worms. ***, P<0.001 (t-test). (TIF) [file pgen.1004699.s004.tif]

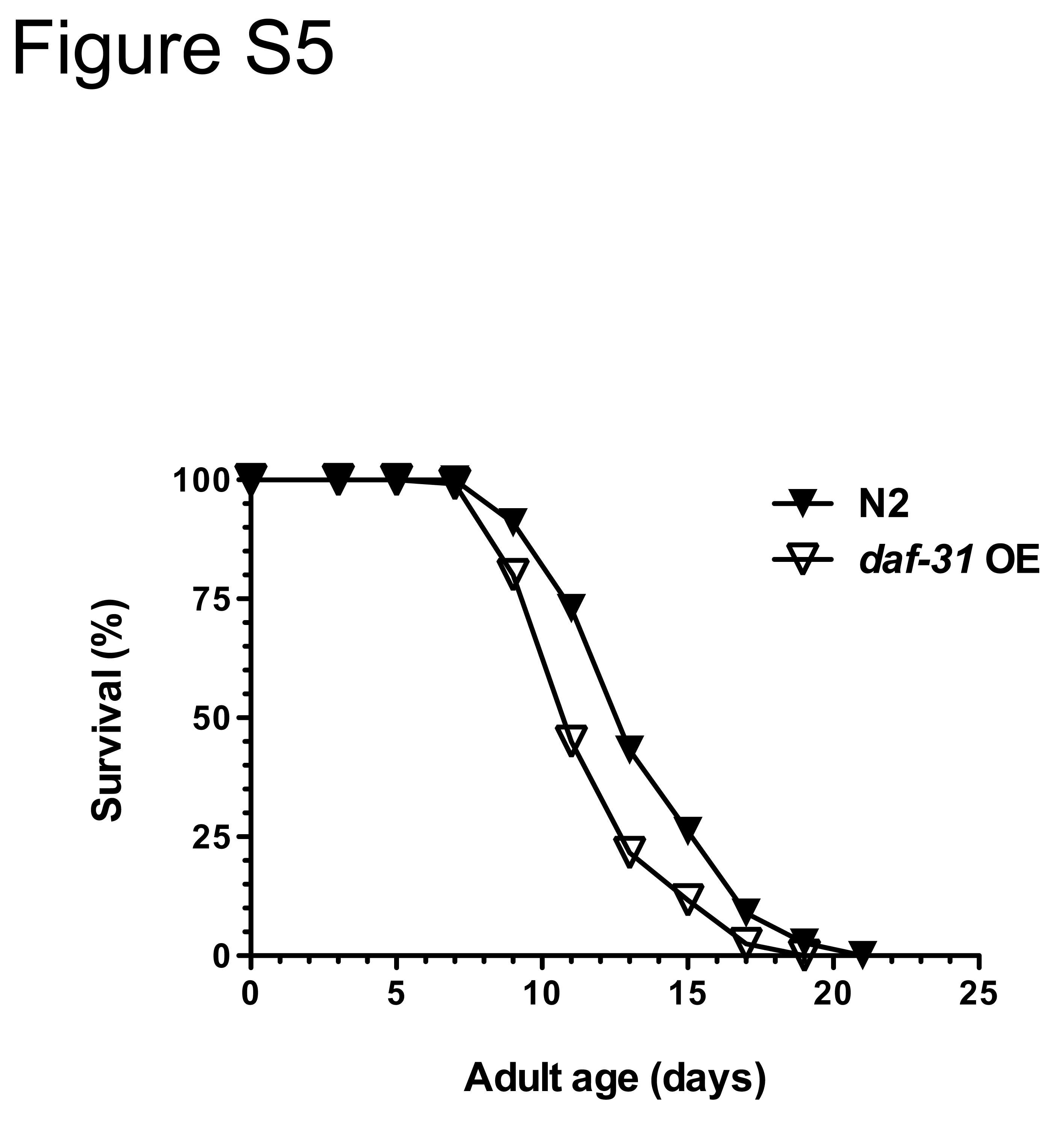

Supplement: Figure S5 — daf-31 overexpression does not extend wild-type C. elegans lifespan. N2 worms and daf-31 overexpressing animals were grown at 20° in the presence of food. L4 hermaphrodites were picked up for lifespan experiments. The statistical analysis of lifespan data is presented in Table S1. (TIF) [file pgen.1004699.s005.tif]

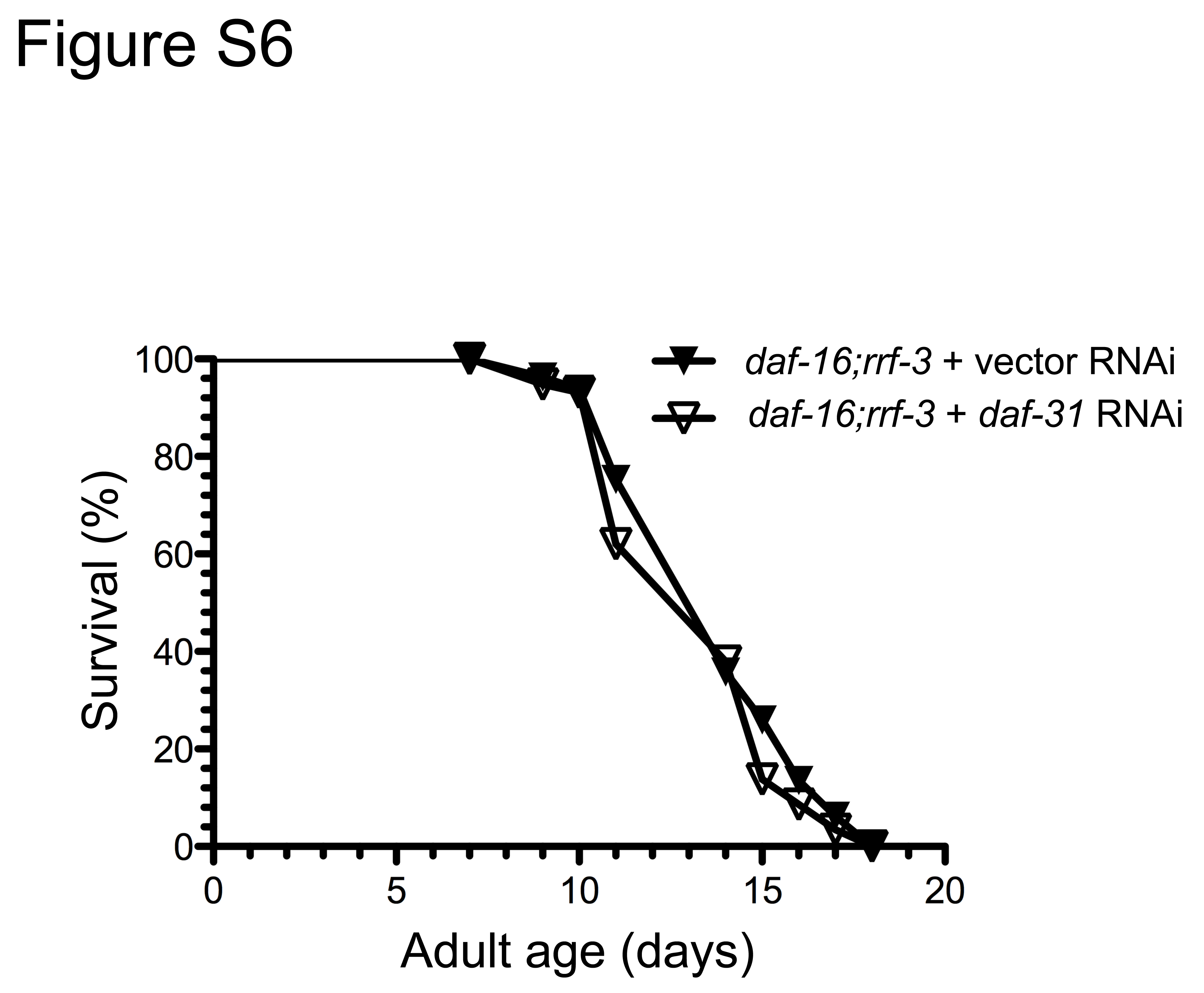

Supplement: Figure S6 — daf-31 RNAi does not influence the lifespan of RNAi-sensitive daf-16 mutants. daf-16;rrf-3 animals were fed E. coli that express daf-31 dsRNA or E. coli carrying the empty vector. The lifespan of RNAi-treated progeny were measured at 20°. (TIF) [file pgen.1004699.s006.tif]

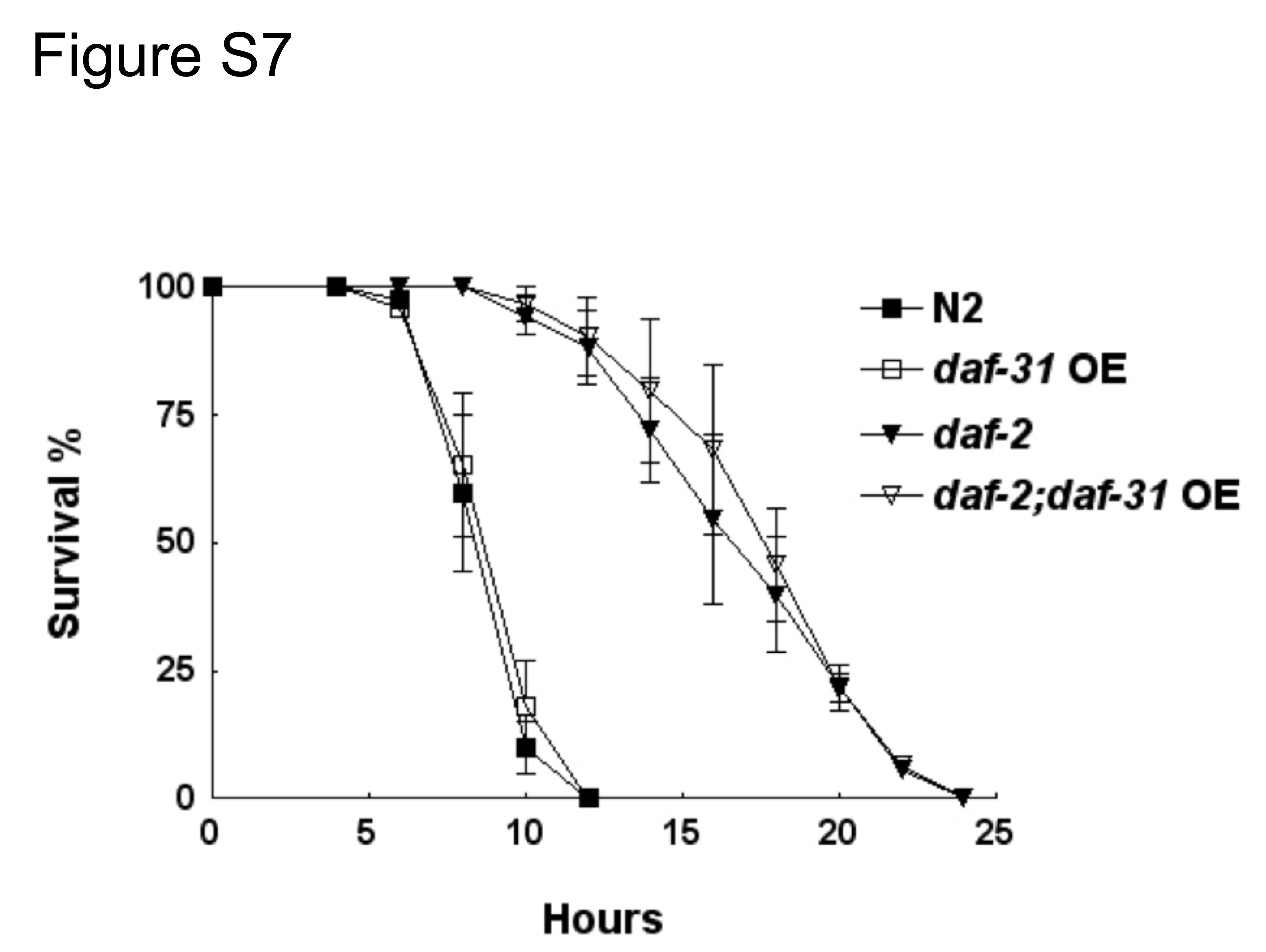

Supplement: Figure S7 — Thermotolerance of animals overexpressing daf-31. daf-31 overexpression does not influence the resistance of N2 worms (p = 0.2420, log-rank test) and daf-2 mutant adults to heat stress at 35°C (P = 0.4623, log-rank-test). (TIF) [file pgen.1004699.s007.tif]

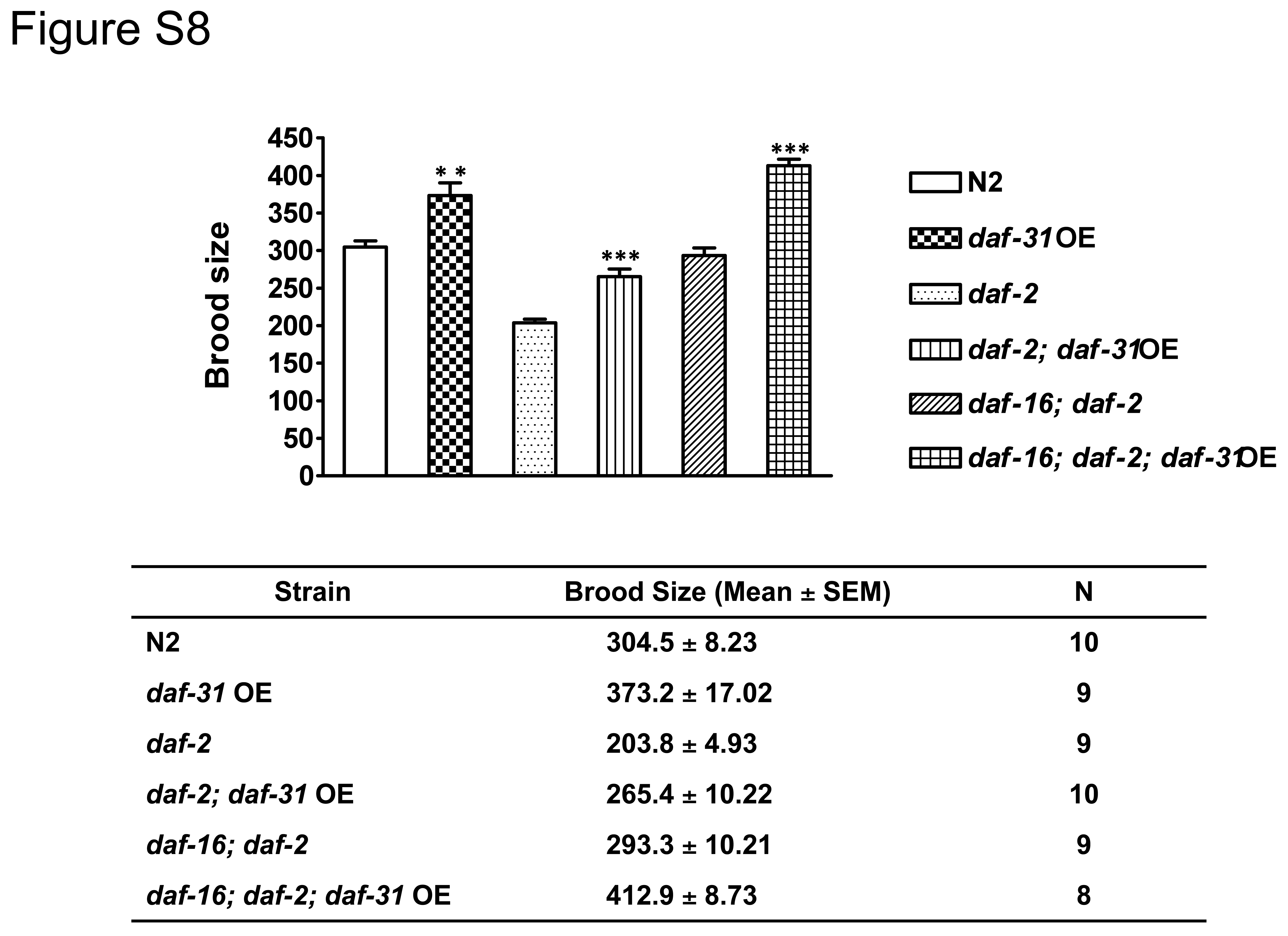

Supplement: Figure S8 — daf-31 overexpression increases reproduction. daf-31 overexpression increases the total number of progeny of N2 and daf-2 mutants, and is not dependent on DAF-16. ** P<0.01, *** P<0.0001 (t-test). (TIF) [file pgen.1004699.s008.tif]

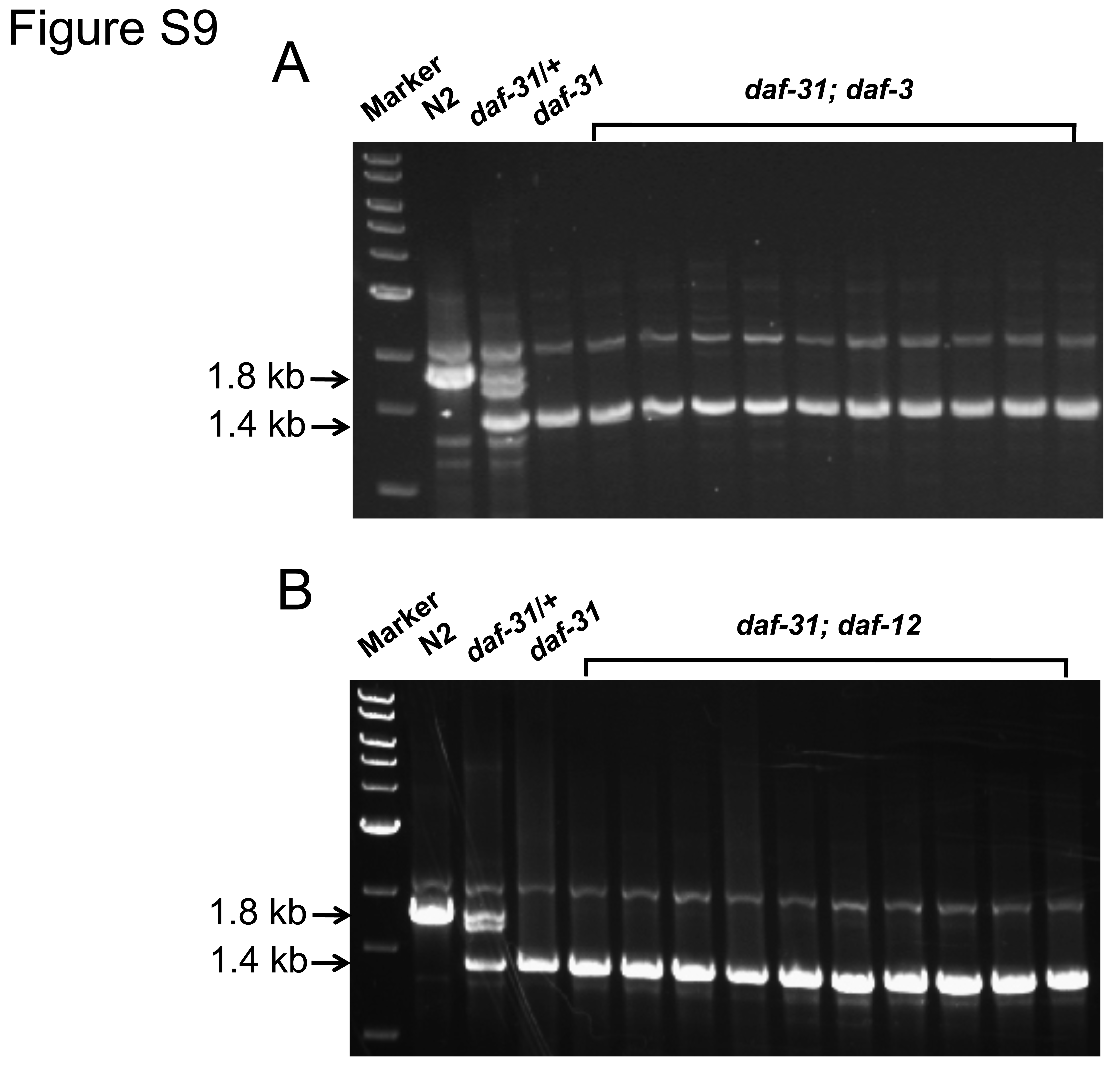

Supplement: Figure S9 — The daf-31 deletion mutation in daf-31;daf-d mutants detected by single worm PCR. Representative gel pictures showing the daf-31 deletion mutation in all daf-31;daf-3 (A) and daf-31;daf-12 (B) homozygous mutants. Arrows indicate the 1,842 bp wild-type band and the 1,449 bp deletion band, respectively. (TIF) [file pgen.1004699.s009.tif]
