# Supplementary material for: daf-31 Encodes the Catalytic Subunit of N Alpha-Acetyltransferase that Regulates Caenorhabditis elegans Development, Metabolism and Adult Lifespan
Source: PLoS Genet. 2014 Oct 16;10(10):e1004699. doi: 10.1371/journal.pgen.1004699 (PMC4199510; doi:10.1371/journal.pgen.1004699)
Supplement: Table S1 — Statistical analysis of daf-31 lifespan data. a Mean lifespan for each trial. b Maximum lifespan for each trial. c Percentage of changes in mean lifespan relative to corresponding control for each trial. d Numbers of animals counted for each trial. e p values (log-rank test) compared to corresponding control. (DOCX) [file pgen.1004699.s010.docx]

**Table S1.** . Statistical analysis of *daf-31* lifespan data

| **Genotype** | **RNAi** | **Lifespan (days)** | | **% of control *^c^*** | **n *^d^*** | ***p* *^e^*** |
| --- | --- | --- | --- | --- | --- | --- |
|  |  | **mean *^a^*** | **max *^b^*** |  |  |  |
| Influence of *daf-31* on the lifespan of wide-type animals | | | | | | |
| N2 | vector | 18.3, 17.5 | 24, 28 | / | 85, 87 | / |
| N2 | *daf-31* | 20.1, 17.2 | 30, 28 | 119%, 98% | 83, 76 | 0.0034, 0.7125, |
| *rrf-3* | vector | 20.1, 19.3 | 26, 27 | / | 87, 49 | / |
| *rrf-3* | *daf-31* | 16.4, 17.4 | 26, 25 | 82%, 90% | 92, 82 | <0.0001, 0.0003 |
| N2 | */* | 12.0, 13.9 | 21, 19 | / | 103, 111 | / |
| N2 *daf-31OE* | */* | 10.9, 12.2 | 19, 17 | 88%, 91% | 117, 120 | 0.0008, <0.0001 |
|  |  |  |  |  |  |  |
| *daf-31* mediates the effect of *daf-2*/IGF pathway on lifespan | | | | | | |
| *rrf-3; daf-2* | vector | 46.4, 47.7 | 66, 66 | / | 74, 72 | / |
| *rrf-3; daf-2* | *daf-31* | 41.2, 42.7 | 62, 60 | 89%, 90% | 74, 63 | 0.0005, 0.0005 |
| *daf-16;rrf-3* | *vector* | 14.0, 14.6 | 18, 19 | / | 81, 26 | / |
| *daf-16;rrf-3* | *daf-31* | 13.4, 13.1 | 18, 21 | 96%, 90% | 58, 34 | 0.2043, 0.1091 |
| *daf-2* | vector | 35.1, 32.7 | 54, 51 | / | 94, 75 | / |
| *daf-2* | *daf-31* | 33.4, 30.6 | 51, 49 | 95%, 94% | 106, 137 | 0.3201, 0.0157 |
| *daf-2; daf-31 OE* | vector | 41.8, 39.1 | 63, 57 | / | 100, 58 | / |
| *daf-2; daf-31 OE* | *daf-31* | 36.2, 30.5 | 54, 49 | 87%, 78% | 118, 143 | <0.0001, <0.0001 |
| *daf-16; daf-2* | / | 11.2, 10.1 | 15, 15 | / | 111, 99 | / |
| *daf-16; daf-2; daf-31 OE* | / | 10.9, 9.8 | 13, 13 | 97%, 97% | 112, 95 | 0.1436, 0.1774 |
