# Supplementary material for: daf-31 Encodes the Catalytic Subunit of N Alpha-Acetyltransferase that Regulates Caenorhabditis elegans Development, Metabolism and Adult Lifespan
Source: PLoS Genet. 2014 Oct 16;10(10):e1004699. doi: 10.1371/journal.pgen.1004699 (PMC4199510; doi:10.1371/journal.pgen.1004699)
Supplement: Table S2 — Statistical analysis of thermotolerance experimental data. a Mean survival for each trial. b Maximum survival for each trial. c Percentage of changes in mean survival relative to corresponding control for each trial. d Numbers of animals counted for each trial. e p values (log-rank test) compared to corresponding control. (DOCX) [file pgen.1004699.s011.docx]

**Table S2.** Statistical analysis of thermotolerance experimental data

| **Strain** | **Survival (hours)** | | **% of control *^c^*** | **n *^d^*** | ***p* *^e^*** |
| --- | --- | --- | --- | --- | --- |
|  | **mean *^a^*** | **maximum*^b^*** |  |  |  |
| N2 | 9.8, 8.9 | 12, 12 | / | 40, 43 | / |
| *daf-31* OE | 10.0, 9.1 | 12, 12 | 102%, 102% | 48, 45 | 0.2420, 0.5353 |
| *daf-2* | 18.6, 16.5 | 24, 24 | / | 45, 42 | / |
| *daf-2; daf-31* OE | 19.3, 17.0 | 24, 24 | 104%, 103% | 46, 58 | 0.4623, 0.5664 |
